# Does deforestation promote or inhibit malaria transmission in the Amazon? A systematic literature review and critical appraisal of current evidence

**DOI:** 10.1098/rstb.2016.0125

**Published:** 2017-04-24

**Authors:** Joanna M. Tucker Lima, Amy Vittor, Sami Rifai, Denis Valle

**Affiliations:** 1 School of Forest Resources and Conservation, University of Florida, 408 McCarty Hall C, PO Box 110339, Gainesville, FL, USA; 2 Department of Medicine, University of Florida, 408 McCarty Hall C, PO Box 110339, Gainesville, FL, USA

**Keywords:** Amazon, *Anopheles*, environmental change, forests, land use / land cover, malaria

## Abstract

Considerable interest in the relationship between biodiversity and disease has recently captured the attention of the research community, with important public policy implications. In particular, malaria in the Amazon region is often cited as an example of how forest conservation can improve public health outcomes. However, despite a growing body of literature and an increased understanding of the relationship between malaria and land use / land cover change (LULC) in Amazonia, contradictions have emerged. While some studies report that deforestation increases malaria risk, others claim the opposite. Assessing malaria risk requires examination of dynamic processes among three main components: (i) the environment (i.e. LULC and landscape transformations), (ii) vector biology (e.g. mosquito species distributions, vector activity and life cycle, plasmodium infection rates), and (iii) human populations (e.g. forest-related activity, host susceptibility, movement patterns). In this paper, we conduct a systematic literature review on malaria risk and deforestation in the Amazon focusing on these three components. We explore key features that are likely to generate these contrasting results using the reviewed articles and our own data from Brazil and Peru, and conclude with suggestions for productive avenues in future research.

This article is part of the themed issue ‘Conservation, biodiversity and infectious disease: scientific evidence and policy implications'.

## 1. Introduction

The idea that environmental change alters the risk of malaria transmission is well established in the literature [1–7]. However, despite a growing body of papers on land use / land cover change (LULC) and malaria in the Amazon (especially related to deforestation), uncertainty pervades our understanding of the relationship between forests, LULC change and malaria in the region. For instance, over the past decade, popular headlines have broadcast contradictory findings: Smithsonian.com declared, ‘Save the Amazon, Increase Malaria’ [8], while ConservationMagazine.org reported, ‘Malaria Linked to Deforestation’ [9]. These claims are based on published scientific articles from Valle & Clark [10] and Vittor *et al.* [11]. As one of the most biodiverse biomes on the planet, conservation of the Amazon rainforests is paramount, but it is critical to determine if conservation has a detrimental or beneficial effect on the health of local populations (i.e. trade-off or win–win scenario between conservation and public health). Depending on the answer to this question, conservation and public policies could benefit from being developed jointly (e.g. to mitigate some of the public health impacts of conservation or to exploit the synergies between these policies), instead of independently as currently done.

Malaria circulates within a complex social-ecological-epidemiological system, and multiple dynamic processes influence transmission risk, requiring careful examination of a diverse set of factors, such as LULC dynamics, mosquito life history and diversity, malaria epidemiology and human behaviour. For example, deforestation [2,11], proliferation of forest edges [12], streams, rivers and standing water along forest margins [13], artificial reservoirs, such as watering holes and aquaculture ponds [14,15] and forest-related activities such as hunting, extraction of forest products (e.g. timber, fruits, medicinal plants), and shifting agriculture [16], have all been blamed for increasing malaria transmission in the Amazon region. Deforestation, in particular, is a common theme in the literature examining the impact of environmental factors on malaria in the Amazon. Still, while some studies conclude that deforestation can reduce malaria transmission [17,18], others claim that forest clearing increases malaria risk [11,19–22]. These contradictions emerge, in part, from differences in definitions, types of data and unstated assumptions regarding the role of forests in malaria transmission. In this paper, we review the current knowledge surrounding deforestation's impact on malaria risk in the Amazon. To this end, we conduct a systematic literature review to characterize and quantify current knowledge on this topic. We then use this review, together with our own data from Acre (Brazilian Amazon) and the Iquitos–Nauta highway (Peruvian Amazon), to identify sources of confusion and themes that require additional research.

## 2. Systematic literature review

For our literature review, we sought out original, peer-reviewed research papers that explicitly address the impacts of deforestation and LULC on malaria in the Amazon region. We used a combination of geographical (e.g. names of countries in the Amazon basin), LULC and malaria-related search terms to query PubMed. Details of our systematic literature search and paper selection are explained in electronic supplementary material, S1, together with a diagram summarizing the filtering process that led to a final list of 47 papers for review. Reviewed articles were published between 1989 and 2015, and most studies originated in Brazil (32). The remaining papers were based in French Guiana (5), Peru (5), Colombia (2), Bolivia (1), Venezuela (1) or covered the entire Amazon region (1). Fourteen papers are entomological studies (30%), 23 are epidemiological studies (49%) and 10 use both entomological and epidemiological data (21%). A complete list of articles returned from our query (with the reasons for exclusion, if applicable) is provided in electronic supplementary material, table S1.

In terms of the relationship between forest cover and malaria, 11% (5 of 47 articles) of the reviewed articles supported a positive association [10,16,23–25], 32% (15 of 47) identified a negative association [11,20–22,26–36] and 26% (12 of 47) found no clear relationship [13,17,37–46]. The ambiguity in the latter cases arose from varying results based on mosquito species in question and the landscape context [42], the type of forest studied (sustainable forest reserve versus protected forest reserve; [45]), or the metric used to measure malaria (entomological inoculation rate (EIR) versus human-biting rate (HBR); [46]). Also, five of these 12 papers supported the idea that initial deforestation in new settlements increases malaria risk, but as deforestation proceeds it can translate into lower malaria risk [13,17,37–39]. Fifteen per cent of reviewed articles (7 of 47) specifically evaluated deforestation *rate* [10,16,25,39,43,47,48], but only three of these found a positive association between deforestation and malaria [39,47,48]. In short, we fail to find overwhelming evidence supporting a consistent simple and straightforward relationship between forests, deforestation rate and malaria.

Importantly, our literature review uncovered substantial differences in method, scale and approach that might help explain how deforestation can both increase and decrease malaria depending on context and study details. We discuss in greater detail the differences that we have found, first focusing on the environment and then focusing on mosquitoes and humans. Within each of these sections, we target aspects that, in our opinion, strongly shape results and conclusions.

## 3. The environment

In exploring the linkages between malaria and forests in the Amazon, different researchers rely on different LULC definitions, LULC classification methods and forest exposure metrics to explain malaria risk, all of which may lead to substantially different conclusions. First, we describe the different definitions for ‘deforestation’ that researchers have adopted. Second, we briefly discuss different approaches to LULC classification and their associated advantages and shortcomings. Third, we highlight the different forest exposure metrics that are commonly employed. Finally, we end this section by discussing the role of water in mediating the relationship between malaria and forests.

### (a) Definitions of ‘deforestation’

We focus on the definition of ‘deforestation’ because considerable confusion surrounds this term in the literature. Forest cover and deforestation are often assumed to represent two sides of the same coin, but they need not be. While 25% (12 of 47) of the reviewed articles define deforestation as the *area* of land cleared of forest (oftentimes measured within predetermined buffer zones around households or mosquito collection sites), deforestation has also been characterized as the *rate* of forest clearing over time (15% of the reviewed articles, 7 of 47). These two definitions describe different concepts—one being static and the other a process. Another 23% (11 of 47) of reviewed articles directly measure forested area, or forest cover. Contrary to intuition, higher deforestation *rates* often occur in areas with high forest cover although this relationship may not be linear.

**Figure 1.**
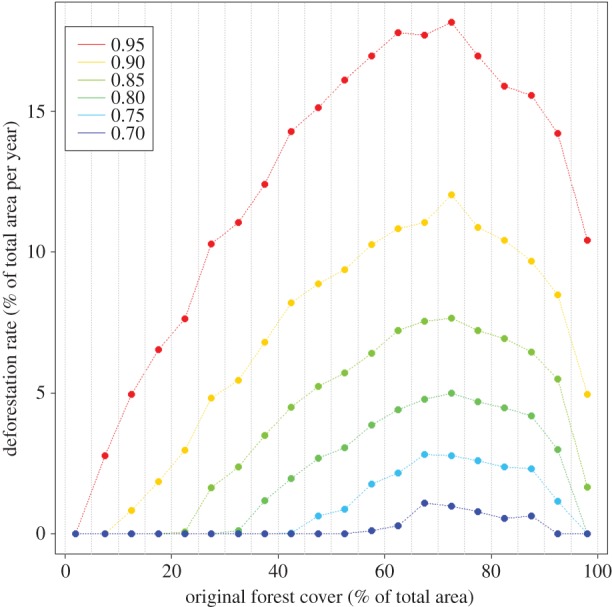
Deforestation rate tends to be higher for properties with substantial forest cover (65–90%) in rural settlement areas in the Amazon region (Acre State, Brazil), decreasing as forest cover is reduced. Original forest cover was discretized into 5% bins (break points are represented with vertical grey lines), and deforestation rate percentiles (0.7 to 0.95) are shown with different colours. Although this figure is based on cross-sectional data, a temporal analysis of the properties that originally had high forest cover reveal the same qualitative pattern.

To illustrate this concept, we consider deforestation rates and forest cover in the Brazilian state of Acre. Using information on individual properties within rural settlement projects in Acre (*n* = 16 188) and annual deforestation maps from 2001 to 2013 based on Landsat imagery (PRODES project, Brazilian Space Agency (INPE)), we find that deforestation rate initially tends to increase for properties with high forest cover and then decrease as forest cover is lost (figure 1). In other words, high deforestation rates tend to be associated with relatively high forest cover, and therefore, a strong association between malaria and deforestation rate may be the result of greater malaria risk in areas with higher forest cover.

Accounts of the historical increase in malaria cases in the Brazilian Amazon region during the late 1970s and 1980s typically attribute this malaria surge to the massive and uncontrolled migration to the region, which led to large-scale deforestation and increased contact of non-immune subjects with forested areas [37,49–52]. Unfortunately, there has been little effort to disentangle the effect of proximity to forest from the effect of deforestation *per se*.

### (b) Land use / land cover classification

How the landscape is characterized can also shape conclusions about the impact of forests on malaria. To estimate deforestation and forest cover, many authors in our review employed remotely sensed imagery (49%, 23 of 47 articles), but level of detail varies substantially among studies [7]. A relatively high percentage of the reviewed papers that use remote sensing relied on a binary classification of LULC (i.e. forest and non-forest; 35%, 8 of 23 articles). This is probably due to the large spatial scale of these studies, which often leads researchers to adopt existing remote sensing products instead of attempting to create a customized LULC classification. For instance, a particularly popular remote sensing product is the yearly deforestation maps provided by INPE's PRODES project (30%, 7 of 23 articles that used remote sensing used these maps). Unfortunately, a binary forest/non-forest classification might be an overly simplistic representation of what is likely to be a continuous vegetation cover gradient [53]. Furthermore, INPE's deforestation maps do not allow for polygons previously classified as deforested to be subsequently classified as forested. As a result, this product fails to capture areas with secondary-forest regrowth that might favour malaria vectors [11,20,29,41].

In our review, 57% of articles that employed remote sensing (13 of 23) identified multiple LULC classes, such as grassland, agriculture and secondary forest. Distinguishing among multiple LULC classes can be important because of potential differences in vegetation structure, shading and luminance (reflected light), which affect mosquito feeding/biting, resting and reproduction (i.e. larval habitats) [2,40]. However, allowing for multiple LULC classes also has its perils. First, researchers seldom use the same LULC categories, hampering the comparison of results from different studies. Second, the same LULC can be labelled in multiple ways, again complicating comparison among studies and leading to substantially different conclusions. For instance, an LULC class with a moderate amount of vegetation may be labelled shrub/low-vegetation or secondary-growth forest. The first label can be interpreted as vegetation that results from natural processes (e.g. flooding or low soil fertility), while the second label implies that the area was deforested and vegetation is re-growing.

**Figure 2.**
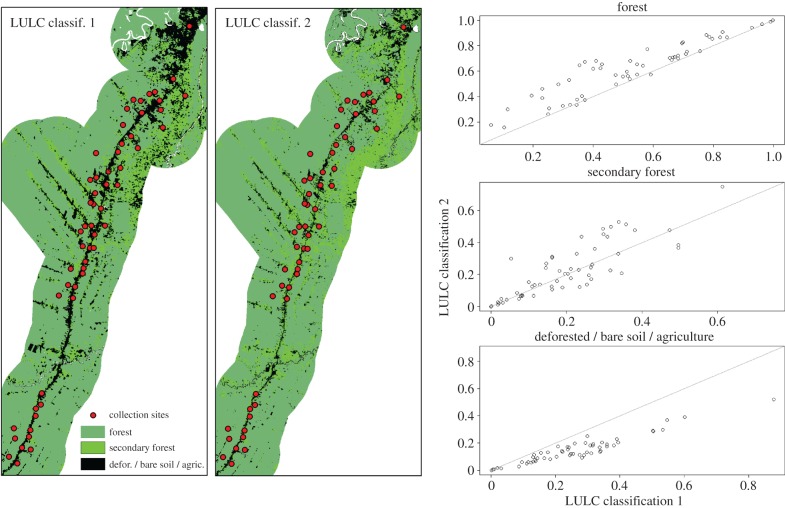
Overall agreement of LULC classification at the landscape level along the Iquitos–Nauta highway (Peruvian Amazon), but with substantial variation in the proportion of each LULC category within 1 km of each adult mosquito collection site. The ‘deforested / bare soil / agriculture’ class represents the sum of the categories ‘impervious area’ and ‘deforested area’ for LULC classification 1 and the sum of the categories ‘agriculture and NPV’ and ‘urban and soil’ for LULC classification 2. The ‘forest’ class consists of the ‘forest’ class in LULC classification 1 and the sum of the ‘terra firme forest’ and ‘flooded forest’ classes in LULC classification 2. A 1 : 1 line was added for reference in scatter-plots (grey line).

Third, researchers use a plethora of methods to classify LULC. While these methods may yield similar results at the landscape level, as judged by overall measures of classification accuracy (Kappa index and overall percentage of cases correctly allocated), specific LULC classes might be poorly predicted and relatively large discrepancies may arise in LULC transitional areas, such as those close to human settlements where most malaria studies are conducted. To illustrate some of these issues, we compare LULC classifications from the Peruvian Amazon performed by two independent research groups (figure 2). The first LULC classification was based on an unsupervised classification of a 2001 Landsat image, which resulted in seven classes: clouds, cloud shadow, forest, secondary forest, water, impervious area and deforested area. The second LULC classification was based on a supervised classification using the Random Forest algorithm on a 2000 Landsat image and resulted in six LULC categories: terra firme forest, flooded forest (‘varzea’), secondary forest, agriculture and non-photosynthetic vegetation (NPV), urban and soil, and water. A comparison reveals overall agreement at the landscape level (left and middle panels) but substantial variation in the proportion of the different LULC categories within 1 km of each adult mosquito collection site (right panels).

Going beyond a binary forest/non-forest classification is important because the LULC that follows deforestation may affect suitability for vectors and malaria transmission [3]. Deforested areas represent a spectrum of realities [7], and identification of the land use or cover replacing the forest can be just as important as the removal of the forest itself. In many remote areas of the Amazon, after landowners clear the original forest and cultivate crops, the area is often abandoned after a few years due to decreased soil fertility, eventually being covered by secondary-growth forest. From our review, 80% of articles that classified secondary forest separately (8 of 10) identify a positive link between malaria and secondary forests that regrow after cycles of deforestation and shifting agriculture. Areas with low-level vegetation (i.e. early secondary-forest growth or taller, bushy crops) are assumed to be critical as *An. darlingi* resting spots and shaded refuges between larval habitats and households [2,11,20,40,54]. Barros & Honório [13] also reported high densities of *An. darlingi* larvae in water collections bordered by secondary forest and tall grasses, and Barbieri *et al.* [55] found a positive correlation between clearing of regrowth forests and an increase in malaria prevalence.

### (c) Metrics of forest exposure

The literature reviewed here utilizes different metrics to assess the impact of forests on malaria risk. For instance, several studies rely on the proportion of different LULC categories within a particular distance of mosquito collection sites (11 of 47 articles). Distance between households and a particular LULC class (e.g. forest, water bodies and secondary forest) is also commonly used to assess the influence of the landscape on malaria risk (16 of 47 articles). The idea that proximity to forest affects malaria incidence is a common thread throughout the literature on forests and malaria (13 of 47 articles). Eight publications from our systematic review specifically measure and test the idea that proximity to forest or forest fringe influences malaria risk. Seventy-five per cent of these (6 of 8) report an increase in malaria incidence or HBR with greater proximity to forest (or alternatively, malaria decreases with increasing distance to forest).

In many of these studies, deforestation is expected to initially increase malaria because it reflects enhanced vector–human contact in forested areas of new human settlements (assuming forests represent prime vector habitat); however, ongoing deforestation tends to increase the distance between households and the forest, which in turn is expected to reduce human contact with malaria vectors [13,17,37–39]. Distinguishing between initial deforestation for human settlement and ongoing deforestation in already established areas is likely to be critical to properly understand how deforestation relates to malaria risk. Forest fringes by themselves have also increasingly received attention as important vector habitat. Barros *et al.* [40] describe the forest fringe as a unique ecotone where larvae tend to cluster, serving as a potential source of malaria vectors. These authors found significantly more *An. darlingi* larvae in forest fringes than in primary forest or deforested sites. Thus, increasing the distance to these transitional zones is expected to lower the risk of contact with malaria vectors. Barros & Honório [13] also reported the absence of *An. darlingi* larvae from open sunlit deforested landscapes, and where forest cover was experimentally removed above and around water bodies, larval clusters disappeared. Forest clearing exposes mosquito larval habitats to sunlight, subsequently reducing larval populations of certain species [2,40].

Another metric often used to determine the association between forests and malaria in epidemiological studies consists of participation in forest-related activities. If malaria risk is high in or near the forest, participation in forest-related activities is expected to increase malaria risk. Fifteen per cent of papers in our review (7 of 47) report that forest-related activities (e.g. land clearing, logging, extraction of forest products, hunting and fishing) increase human contact with malaria vectors leading to a rise in malaria cases, but only 57% of these (4 of 7) collected survey data to test this relationship [16,19,56,57]. The association between forest-related activities and malaria is supported by Bauch *et al.* [45], who found that strictly protected forest areas promote lower malaria transmission, while sustainable-use protected areas are associated with higher malaria transmission. On the other hand, Barros *et al.* [58] and Silva-Nunes *et al.* [19] found no evidence that activities like hunting or fishing, which involve sleeping away from home (outside), increase malaria risk. Conflicting findings about the connection between forest-related activities and malaria incidence suggest that the unique qualities of the forest under study and the particular activity undertaken are important. Forest characteristics such as level of degradation, degree of human disturbance and openness of the forest canopy (e.g. penetration of sunlight to the ground level, where water accumulations support potential aquatic habitats for mosquitoes) are difficult to assess but can provide information relevant to vector biology and in turn, malaria risk [43].

**Figure 3.**
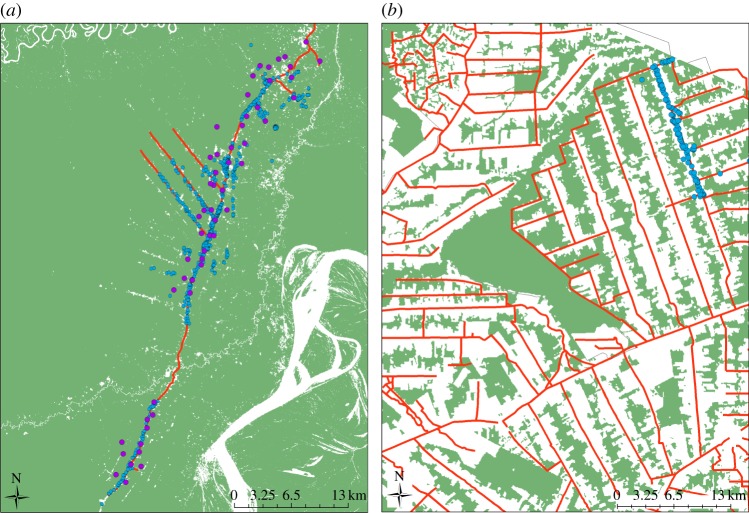
Contrasting landscape-level deforestation in studies focused on the relationship between malaria and LULC change. (*a*) Landscape along the Iquitos–Nauta highway (Peru) in 2001 in Vittor *et al.* [11,20]. (*b*) Landscape in the rural settlement project Pedro Peixoto (Acre, Brazil) in 2004 in Silva-Nunes *et al.* [19] and Valle *et al.* [16]. Red lines are roads, green polygons are forest and blue points are households (left: households enumerated by Vittor *et al.*; right: households that participated in the Pedro Peixoto study). Purple points are locations where vector data were collected. Note that no vector data were collected for the Pedro Peixoto study. Both maps are in the same spatial scale.

Finally, landscape-level characteristics might also be critical in determining the relationship between forests and malaria. For instance, although both Vittor *et al.* [11,20] and Valle *et al.* [16] focused on the role of deforestation on malaria, the landscapes within which these studies were conducted were radically different, being at almost opposite ends of the forest cover spectrum (figure 3). Conceivably, the removal of forest in a highly deforested landscape has a substantially different effect to removal of forest in a highly forested landscape. Furthermore, some authors specifically identify the configuration of forests and other LULC classes over the landscape as an important driver of malaria risk (3 of 47 reviewed articles). For instance, it has been suggested that a landscape composed of more diverse LULC categories provides more potential habitat for malaria vectors (even for multiple-vector species), both as adults and as developing larvae [24,33,41]. A higher degree of landscape division (i.e. probability that two randomly chosen places in the landscape under investigation are not situated in the same undissected area; [59]) stimulates higher densities of malaria vectors [41] and is correlated with malaria incidence in children [24]. An increasingly fragmented landscape is also likely to expose more forest fringe (figure 3), which may help explain higher malaria incidence in areas with higher levels of landscape division than in less fragmented areas.

### (d) Role of water

The relationship between forest and malaria risk is likely to be mediated by yet another key landscape feature: water. Female *Anopheles* mosquito vectors alternate between seeking a blood-meal and oviposition in water bodies, thus water is critical to the mosquito's life cycle and malaria transmission [60]. Given the climatic and environmental circumstances that support mosquito survival, flight distances between water and a meal determine vector abundances and distributions, and the prominence and configuration of water on the landscape can have a significant impact on malaria risk. When landowners deforest for the express purpose of installing fish ponds or damming of waterways to form watering holes for cattle, habitat for mosquito larvae (specifically *An. darlingi*) expands, as long as some vegetation is present in the water or along the water's edge to provide shade [13]. The formation of fish ponds for aquaculture may be a largely overlooked driver of elevated malaria transmission in deforested areas [13]. Microdams formed when fallen trees obstruct streams and rivers also provide prime larval habitat for malaria vectors. According to Barros & Honório [13], proximity of houses to water bodies with low luminance can be more important to malaria risk than proximity to forest fringe itself. Still, Barros *et al.* [40] registered significantly higher densities of microdams in forest fringe areas.

Twenty studies in our review (43%) included either per cent water cover or distance to water bodies as a covariate. Among these 20 articles, eight articles report a positive relationship between malaria and water [11,13,32,40,48,58,61,62] while results from the remaining 12 articles were inconclusive. One of the key challenges in determining the influence of water on malaria risk is that many of the water bodies containing *An. darlingi* larvae are too small to be detected by remote sensing [20]. Even if they are large enough, vegetation may cover the water bodies, making their detection through optical remote sensing (i.e. non-radar) difficult. This is important because even if water bodies in deforested areas are more likely to harbour *An. darlingi* larvae [20], the overall abundance of water bodies in deforested versus forested landscapes is relevant to determining how these findings translate into higher numbers of *An. darlingi* adult mosquitoes [63]. Finally, field data collection on water bodies can be complicated. For example, how does one estimate the area of (or distance to) a meandering creek or stream or multiple very small water bodies (e.g. animal hoof prints) in a feasible manner? Another challenge refers to the variety of types of water bodies (e.g. deep-water ponds, fishing farms, dammed streams, seasonally flooded areas, or river banks), which vary substantially in terms of their suitability as mosquito aquatic habitat [14,15,61].

## 4. Mosquitoes and humans

The above considerations regarding landscape features and perceptions about the role of forests and deforestation in malaria transmission have limited value in the absence of information on malaria vector biology/ecology [64]. Understanding mosquito abundance, diversity and life cycles as related to the environment, as well as the capacity of different mosquito species to infect human populations with malaria parasites (*Plasmodium* spp.), facilitates a deeper understanding of how deforestation and its outcomes impact malaria incidence.

In our review, 30% (14 of 47) of the articles focused exclusively on entomology. Interestingly, despite the recurring claim that the rise of mosquitoes and malaria primarily results from an increase in aquatic habitats after forest clearing, relatively few studies have specifically collected comprehensive data on mosquito larval distributions. Nine out of 24 reviewed studies that included entomological data (38%) collected larval samples, whereas 88% of studies that use entomological data (21 of 24) collected adult mosquitoes.

Ample evidence demonstrates that environmental alterations, like deforestation, affect mosquito populations, both in terms of abundance and species composition [27,33,46]. However, each mosquito species has unique life-history characteristics and habitat preferences, and therefore reacts differently to landscape changes. This is important because although *An. darlingi* has long been acknowledged to be the main malaria vector in Amazonia [65], other mosquito species have also been found to be important in local transmission of malaria in the region, including *An. nuneztovari* [27,41], *An. marajoara* (a member of the *An. albitarsis* complex) [28,66,67], *An. albitarsis* [68,69], *An. benarrochi* [70], *An. oswaldoi*, *An. trinkae* and *An. albimanus* [71].

**Figure 4.**
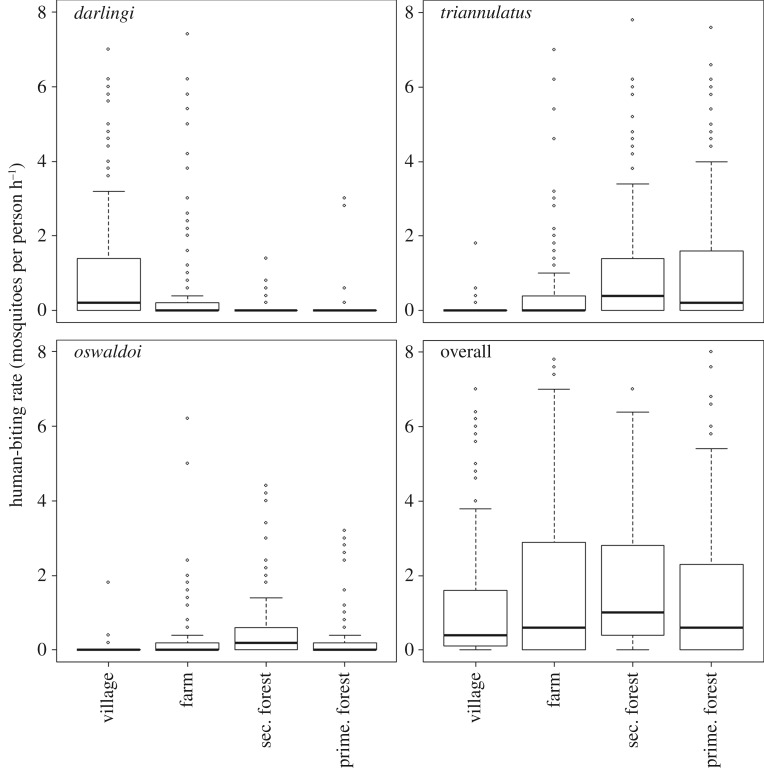
Distinct LULC preferences for different mosquito vector species. Human-biting rate (HBR) for the three most abundant Anopheline species at our study site along the Iquitos-Nauta highway in Peru (i.e. *An. darlingi*, *An. triannulatus* and *An. oswaldoi*), according to different LULC as assessed in the field. The lower right panel shows the overall human-biting rate, calculated as the sum of the biting rate from *An. darlingi*, *An. triannulatus*, *An. oswaldoi*, *An. nuneztovari*, *An. benarrochi* and *An. rangeli*. Note that the *y*-axis was truncated at 8 for clarity. The maximum human-biting rate was equal to 19.6, 61.6, 24.8 and 61.8 bites per 6 h (6pm to midnight) for *An. darlingi*, *An. triannulatus*, *An. oswaldoi* and overall, respectively. Data collection was restricted to this 6 h period because a pilot study determined that *An. darlingi* HBR peaks between 21.00 and 23.00 [11], but ideally a comparison of HBR among species would be based on data collected all night [72].

To understand the role of deforestation on malaria risk, it is important to consider the impact of LULC changes on these different species. To illustrate this, we compare human-biting rates of the three most abundant mosquito vector species collected at our study site in the Peruvian Amazon across four LULC classes (figure 4). Displaying the highest HBR, *An. triannulatus* tends to bite more in forested areas, whether primary or secondary forest, and neither this species nor *An. oswaldoi* are common in village sites. *An. darlingi* showed the opposite pattern, most frequently found biting in the village and rarely in forested areas. This complementarity reveals the complexity of trying to associate a particular land cover or land use with malaria. Studies focused solely on a single anopheline species (e.g. *An. darlingi*) may arrive at different conclusions to studies that include multiple-vector species.

Most studies accept the premise that malaria is contracted primarily in and around the home (but see [41]), even though in most parts of its vast range, *An. darlingi* tends to rest and feed outdoors (P Lounibos 2016, personal communication; see review in [73]). As a result, the majority of entomological studies reviewed here focus mosquito sampling efforts outside the forest, whether in terms of biting rate, adult mosquito capture or larval surveys: 58% of studies collected mosquitoes in peridomestic areas and/or indoors (14 or 24). Unfortunately, this leads to a circular logic where mosquito sampling in peridomestic areas or indoors leads to higher estimates of *An. darlingi* abundance, which reinforces the notion that this mosquito species is the main malaria vector in the region [42]. This conclusion then leads studies on the relationship between LULC and malaria to focus solely on *An. darlingi*, which then confirm that *An. darlingi* is found in more disturbed environments [27], such as areas close to households. Seven of the 24 articles that collected entomology data (29%) focused exclusively on *An. darlingi*.

It is also important to acknowledge that a higher HBR for a particular mosquito species does not necessarily translate into higher malaria risk. First, substantial species differences may exist regarding other key Vectorial Capacity parameters [74], such as female mosquito longevity (which is critical given the need of female mosquitoes to feed on humans to acquire gametocytes, to complete the extrinsic incubation process, and then to transmit sporozoites to humans) [23,75] and the proportion infected with the malaria plasmodia. Conn *et al.* [28] found significantly more *An. marajoara* mosquitoes infected with *Plasmodium* spp. than *An. darlingi* at a study site in the Eastern Amazon. Infection by *P. falciparum* and *P. vivax* also varies by mosquito species. For instance, while *P. falciparum* was predominantly found in *An. darlingi*, in some instances *P. vivax* sporozoites were found at greater frequencies in *A. triannulatus, A. nuneztovari* and *A. albitarsis* than in *A. darlingi,* suggesting that the former three species probably play an important role in the transmission of *P. vivax* malaria [76]. Unfortunately, determining the robustness of these results is difficult because *Plasmodium* spp. infection rates in mosquitoes are very low, generally between 0.1 and 3.7% [27,34,35,41,77], and because an enzyme-linked immunosorbent assay (ELISA) was used, which is known to generate false-positives [78] and is not as sensitive as microscopic techniques [79]. Furthermore, while it may be tempting to attribute these results to differences in the species' inherent ability to carry and transmit malaria plasmodia (i.e. vector competence), this may not be the case. For these reasons, experimental infection studies of distinct *Anopheles* species are important (e.g. [80]).

Second, people visit different LULC categories at different times of the day and spend different amounts of time in each place. As a result, exposure to different mosquito species varies considerably and HBR *per se* might not be enough to determine the primary malaria vector. For instance, HBR might be high in agricultural fields based on night captures but that may be inconsequential if people only visit their agricultural plots during the day. Similarly, people may spend so much time in their villages at night that, despite the lower HBR, this LULC category is where most malaria transmission occurs. Finally, some people sleep under bed nets at home but readily expose themselves to mosquitoes when going fishing or hunting at night in secondary/primary forest. Given that no method exists to determine when and where infection occurs, understanding human behaviour becomes critical in determining the ultimate role of different mosquito species as malaria vectors. Unfortunately, similar to most entomological studies, epidemiological studies typically correlate the environmental characteristics surrounding each household to infection probability, with the implicit assumption that infection occurs in the house or its vicinity.

In relation to epidemiological data, although a majority of reviewed papers considered epidemiological evidence (70%; 33 of 47), most of these studies analysed secondary data (i.e. data from health facilities; *n* = 23). This can be limiting, as people only seek help at health facilities if they feel sick, and therefore these data fail to capture asymptomatic carriers. This, in turn, can bias conclusions regarding forest cover and malaria, given that people residing longer in the region are more likely to live in deforested areas and to develop clinical immunity [19,81]. Secondary data are also frequently aggregated at the health facility or county level. While studies relating forest cover, deforestation rate and malaria using aggregate data are useful, statistical associations established with aggregate data are notorious for their precariousness (e.g. these associations may reverse signs when data are disaggregated to the individual level, a phenomenon widely known as ‘ecological fallacy’ [82]). Furthermore, substantial differences in sampling effort (e.g. number of health facilities) between counties may confound results when analysing county-level malaria incidence data.

Finally, some studies suggest that *An. darlingi* abundance is more a function of human presence than disturbance itself [30,42]. For instance, even though about 10 times more mosquitoes (from multiple anopheline species) were captured within dense forest than in a nearby village in Peru, 300 times more *An. darlingi* were captured in the village compared with the forest [30]. The village and forest collection sites were separated by less than 300 m, and the authors attribute this stark difference in vector abundance to the availability of human blood meals, as opposed to differences in vegetation cover, or deforestation. On the other hand, Vittor *et al.* [11] compared levels of forest cover, deforested areas and secondary vegetation, finding that *An. darlingi* was more frequently captured at sites with little remaining forest, even after controlling for human presence. More studies are needed to better disentangle the effects of forest cover from that of human presence for multiple-vector species.

## 5. Conclusion and recommendations for future study

In this article, we have reviewed the literature on the relationship between deforestation and malaria in the Amazon region and have highlighted topics that contribute to the disparate findings reported in the literature. We believe that the diversity of topics covered in this article testifies to the fact that this apparently simple problem can only be tackled by highly interdisciplinary research teams involving medical entomologists, malaria epidemiologists, LULC/remote-sensing specialists and anthropologists.

**Figure 5.**
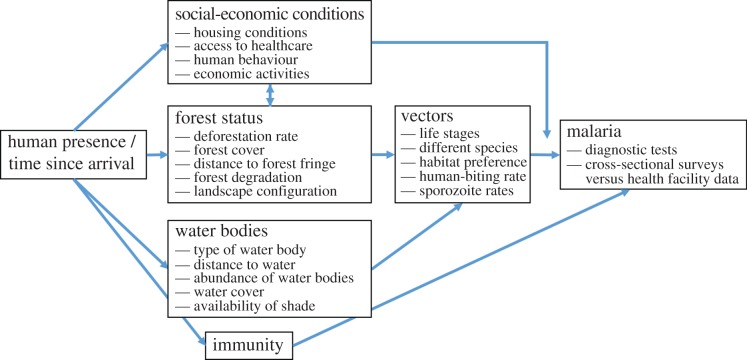
Causal diagram for the relationship between forest status and malaria. Within each box, we list the characteristics/metrics associated with each of these topics. Arrows indicate likely causal relationships, which in some cases may be bi-directional (e.g. between forest status and social-economic conditions). We represent the fact that social-economic conditions mediate the relationship between vectors and malaria (e.g. through changes in housing conditions and access to healthcare) by having its arrow pointing to another arrow. (Online version in colour.)

We summarize our conceptualization of how the different drivers of malaria risk are related to each other and to malaria using a causal diagram (figure 5). Although this is admittedly an incomplete representation of reality, we hope that the causal diagram is useful in highlighting the main malaria drivers and their inter-relationships. Based on this diagram, we provide several suggestions regarding how to move the field forward. Studies that integrate entomological and epidemiological data are critical to understand how environmental changes (e.g. deforestation) influence malaria vectors and how these changes ultimately impact malaria risk. Unfortunately, these studies are still relatively rare, comprising 21% (10 of 47) of the papers in our review. An ideal study would devote significant effort to understanding the environment, vectors and humans and how they interact. In relation to the environment, a good characterization of different vegetation types (e.g. degraded forests, secondary and primary forests, etc.) and types of water bodies (e.g. fish ponds, streams, hoof prints) is critical. The monitoring of multiple mosquito species and life stages (i.e. larvae and adult mosquitoes) is also likely to be important in generating a more mechanistic understanding of how the environment influences mosquito populations. Besides examining larval habitats, entomological studies should pay close attention to characterizing mosquito resting sites, as this is an often cited (but under-studied) mechanism through which vegetation influences adult mosquito presence. As for humans, assessing malaria prevalence is likely to require cross-sectional surveys where all individuals are tested with very sensitive and specific diagnostic methods (e.g. polymerase chain reaction (PCR)). This is critical as several locations in the Amazon region have been shown to harbour a large fraction of infected but asymptomatic individuals that because of their low parasitemia are often only detectable using molecular methods such as PCR [19,83,84]. Longitudinal epidemiological studies employing active surveillance would be especially useful for elucidating the temporal dynamics underlying ecological change and malaria transmission. Finally, the monitoring of human behaviour (e.g. movement patterns) will also be crucial in relating malaria risk to LULC and entomological findings, and new technologies such as GPS loggers [85–87] are likely to be play an important role in minimizing recall bias.

Our diagram also emphasizes how the association between forest status and malaria can be confounded with multiple factors such as water bodies, social-economic conditions and immunity. For instance, in the absence of information on the vectors, observational studies cannot adequately explain how forest status influences malaria because forests impact the vector, are associated with certain socio-economic characteristics (e.g. people in areas with high forest cover typically live in houses in worse conditions and do not have access to healthcare), and are indirectly associated with immunity (i.e. long-term residents in the region tend to live in more deforested areas and to have clinical immunity to malaria). Therefore, the ideal study needs to rely on carefully designed experiments to disentangle these effects. For instance, experimental manipulation of vegetation cover (e.g. as performed by [13]) and water could provide important insights regarding the drivers of mosquito presence and abundance. Similarly, experimental screening of houses could help determine the relative importance of indoor versus outdoor infections, whereas aggressive insecticide spraying indoors and in peridomestic areas for households involved in forest activities might reveal the proportion of infections associated with these forest activities. Finally, an experiment where stagnant water bodies in close proximity to human dwellings are drained may reveal the role of these water bodies on overall malaria incidence. Unfortunately, household-level experiments to assess how changes in forest cover are directly associated with malaria risk, while keeping the other conditions (e.g. socio-economic status and immunity levels) the same, are much more challenging to implement. For instance, one approach consists in randomly choosing households to receive payment for ecosystem services, this way potentially decreasing deforestation rates and resulting in higher forest cover around these households. The problem with this experimental set-up is that this payment also induces changes in income and economic activities, which may ultimately confound the experimental results.

It has long been acknowledged that social and economic variables are highly associated with malaria risk, such as participation in logging and mining activities, population density, better access to healthcare and improved housing conditions [25,39,55,88–90]. Owing to space constraints, our review has admittedly neglected several of these factors and focused almost exclusively on the physical environment and how it relates to mosquitoes and malaria risk. Nevertheless, we believe this review is useful for researchers interested in exploring the relationship between deforestation and malaria in the Amazon region. The Amazon is known for its high biodiversity and relatively pristine environment, but at the same time it is also characterized by very poor socio-economic conditions (e.g. poverty, education, infant and maternal mortality), including serious health problems such as malaria [91]. Given that multiple large-scale development projects are underway in the region (e.g. the Initiative for the Integration of Regional Infrastructure in South America (IIRSA)) and that deforestation is probably to continue into the future [92], a better understanding of the interdependency between deforestation and human health will remain a vital and urgent research theme.

## Supplementary Material

Supplementary Material I. Details of systematic literature review

## Supplementary Material

Supplementary Table I: List of articles from search in PubMed

## Supplementary Material

Supplementary Material II. Entomological data from Peru
