# Supplementary material for: Does deforestation promote or inhibit malaria transmission in the Amazon? A systematic literature review and critical appraisal of current evidence
Source: Philos Trans R Soc Lond B Biol Sci. 2017 Apr 24;372(1722):20160125. doi: 10.1098/rstb.2016.0125 (PMC5413873; doi:10.1098/rstb.2016.0125)
Supplement: Supplementary Material I. Details of systematic literature review [file rstb20160125supp1.pdf]

Supplementary Material I. Search terms, selection criteria and filtering process used in systematic review of literature on the impacts of deforestation and land use land cover (LULC) on malaria in the Amazon region.

We performed a literature search in March 2016 using PubMed, an online database from the US National Library of Medicine and National Institutes of Health with over 25 million citations from the biomedical literature. The following search terms were used to query Title/Abstract fields within the PubMed database:

> (Amazon* OR Brazil OR Bolivia OR Colombia OR Ecuador OR Guiana OR Peru OR Suriname OR Venezuela)
>
> AND
>
> (Deforestation OR Forest OR Land use OR Land cover OR Frontier)
>
> AND
>
> (Malaria OR Anophel* OR Plasmod*)

This query returned 182 publications (see Supplementary Table I). Next, we filtered articles according to the following criteria:

1. Study performed within the Amazon region
2. Study deals specifically with human malaria
3. Study addresses the topic of land use/land cover (LULC)
4. Study is a peer-reviewed research article

We only retained studies with primary or secondary data (i.e., we excluded published literature reviews, editorials, government reports, and/or commentaries). Furthermore, we excluded articles not written in English or Portuguese. The diagram below summarizes the filtering process, which led to a total of 47 articles that met the above criteria and were selected for review.

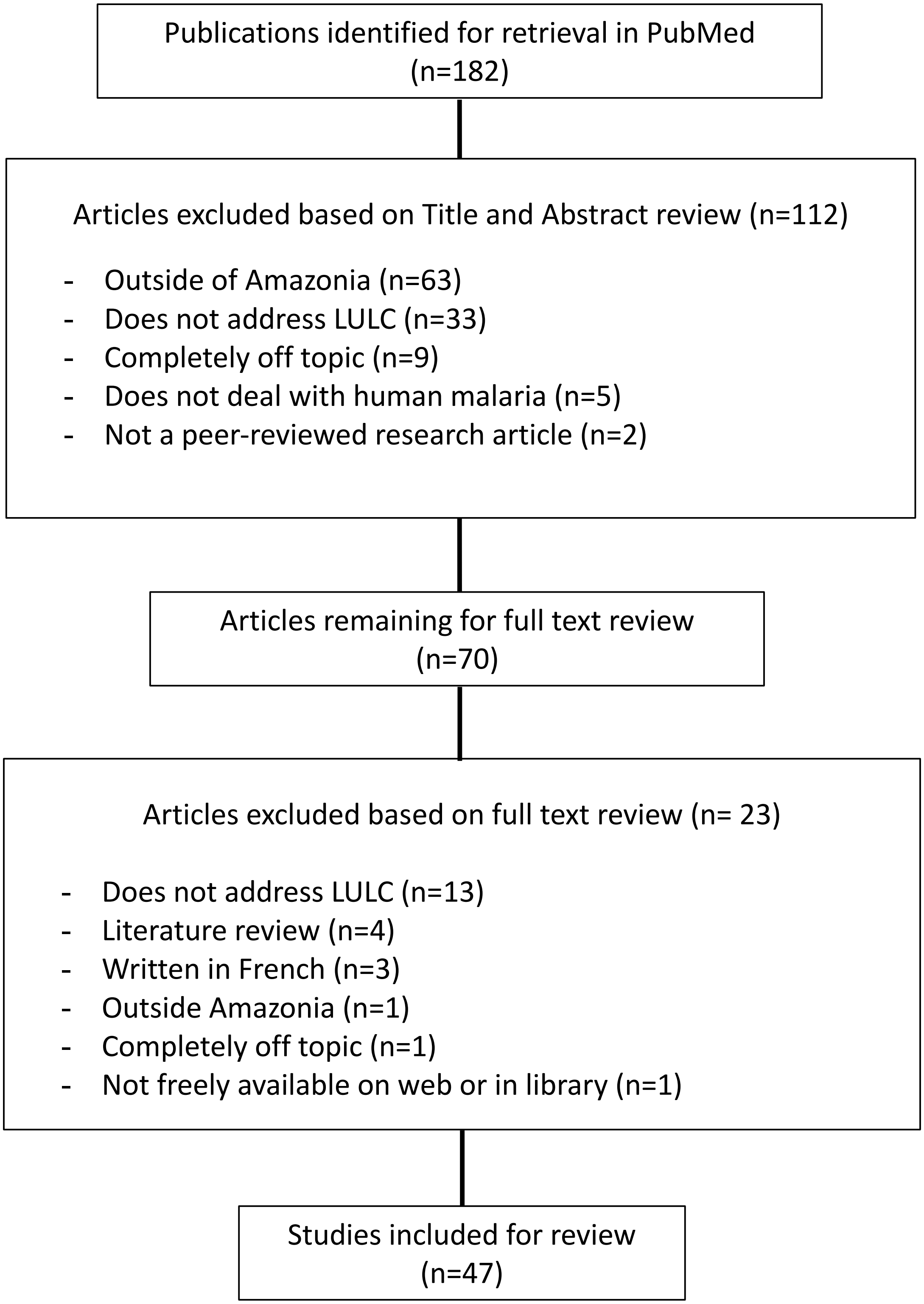
Publications identified for retrieval in PubMed (n=182)
Articles excluded based on Title and Abstract review (n=112)
- Outside of Amazonia (n=63)
- Does not address LULC (n=33)
- Completely off topic (n=9)
- Does not deal with human malaria (n=5)
- Not a peer-reviewed research article (n=2)
Articles remaining for full text review (n=70)
Articles excluded based on full text review (n= 23)
- Does not address LULC (n=13)
- Literature review (n=4)
- Written in French (n=3)
- Outside Amazonia (n=1)
- Completely off topic (n=1)
- Not freely available on web or in library (n=1)
Studies included for review (n=47)
